# Cessation of Rectal Screening for Vancomycin-Resistant Enterococci: Experience from a Tertiary Care Hospital from Türkiye

**DOI:** 10.3390/healthcare11192641

**Published:** 2023-09-28

**Authors:** Gülçin Telli Dizman, Gökhan Metan, Pınar Zarakolu, Elif Seren Tanrıverdi, Gülşen Hazırolan, Hanife Aytaç Ak, Dilek Kılınçarslan, Mertcan Uzun, Başak Çelik Kavaklılar, Zafer Arık, Barış Otlu, Serhat Ünal

**Affiliations:** 1 Department of Infectious Diseases and Clinical Microbiology, Faculty of Medicine, Hacettepe University, Ankara 06800, Türkiye; gokhanmetan@hacettepe.edu.tr (G.M.); zarakolu@hacettepe.edu.tr (P.Z.); mertcan.uzun@hacettepe.edu.tr (M.U.); sunal@hacettepe.edu.tr (S.Ü.); 2 Infection Control Committee, Hacettepe University Hospitals, Ankara 06800, Türkiye; hanifea@hacettepe.edu.tr (H.A.A.); dilekbur@hacettepe.edu.tr (D.K.); 3 Molecular Microbiology Laboratory, Department of Medical Microbiology, Faculty of Medicine, İnönü University, Malatya 44280, Türkiye; seren.tanriverdi@inonu.edu.tr (E.S.T.); baris.otlu@inonu.edu.tr (B.O.); 4 Department of Medical Microbiology, Faculty of Medicine, Hacettepe University, Ankara 06800, Türkiye; gcetin@hacettepe.edu.tr; 5 Department of Internal Medicine, Faculty of Medicine, Hacettepe University, Ankara 06800, Türkiye; basakcelik@hacettepe.edu.tr; 6 Department of Internal Medicine, Section of Oncology, Faculty of Medicine, Hacettepe University, Ankara 06800, Türkiye; zafer.arik@hacettepe.edu.tr

**Keywords:** vancomycin-resistant *Enterococci*, rectal screening, surveillance, outbreak

## Abstract

Objective: Here, we compared the impact of different polices on the epidemiology of Vancomycin-resistant *Enterococcus faecium* bloodstream infections (VRE-BSIs) in a tertiary care hospital including two hospital buildings (oncology and adult hospitals) in the same campus. Material and Methods: All patients who were hospitalized in high-risk units were screened weekly for VRE colonization via rectal swab between January 2006 and January 2013. After January 2013, VRE screening was only performed in cases of suspicion of VRE outbreak and during point prevalence studies to evaluate the epidemiology of VRE colonization. Contact precautions were in place for all VRE-positive patients. The incidence density rates of hospital-acquired (HA)-VRE-BSIs were compared between two periods. Results: While the rate of VRE colonization was higher in the second period (5% vs. 9.5% (*p* < 0.01) for the adult hospital, and 6.4% vs. 12% (*p* = 0.02 for the oncology hospital), there was no increase in the incidence rate HA-VRE BSIs after the cessation of routine rectal screening in either of the hospitals. Conclusion: Screening policies should be dynamic and individualized according to the epidemiology of VRE as well as the workforce and cost. Periodical rectal screening of VRE can be discontinued if suspicion of an outbreak can be carefully monitored.

## 1. Introduction

Antibiotic resistance is a significant and growing global problem that poses a serious threat to public health, especially with respect to healthcare-acquired (HA) infections [1]. Vancomycin-resistant *Enterococcus faecium* (VRE) is one of the leading causes of HA infections [2]. Bloodstream infections (BSIs) that are caused by VRE are associated with significant morbidity and mortality. Mortality rates range between 20% and 52% [3,4,5,6,7]. In a study that investigated the epidemiological trends of vancomycin resistance in *Enterococci* isolates from patients with BSIs in Europe, VRE increased from 8.1% in 2012 to 19.0% in 2018 [8]. In Türkiye, *Enterococci* were reported as the causative bacteria of 13.2% of BSIs and 11.2% of central catheter-related BSI in 2021. About half of them were *Enterococcus faecium*, and rates of vancomycin resistance were 17.2 and 20%, respectively [9].

Active surveillance via screening with rectal swabs to detect VRE colonization and implementing contact precautions has been recommended to control and prevent infections for many years [10,11]. This approach has been found useful in outbreak settings in several studies. However, it is less clear if rectal screening-based surveillance helps to control VRE infections. The availability of isolation rooms, and even sometimes of preserved areas for cohorting the patients, are limited in many healthcare settings. Cohorting the staff is also as important as isolating the patients to prevent the spread of VRE by healthcare staff [10,12]. In a recent study conducted in Japan, it was found that the risk of VRE acquisition in susceptible patients who were in the same room as VRE-positive patients and cared for by the same nurses was six times higher compared to patients who were in a different room and cared for by different nurses [13]. Any center which decides to use rectal swabs for VRE screening as part of a routine infection control programme should account for all these factors together.

However, several recent studies showed that relaxing or discontinuing VRE control programs did not result in an increase in VRE infections, particularly in settings where compliance rates for hand hygiene was high [3,14,15,16,17]. In comparison, some studies reported an increase in VRE infections after routine screening was discontinued [18,19].

Periodical VRE screening via rectal swabs was started in 2006 at all high-risk units at our hospital, which was discontinued because of budget and reimbursement problems in 2016. Although efforts at developing a high-quality infection prevention and control (IPC) policy were first implemented in the early 1980s at our hospital, the average rate of hand hygiene compliance, particularly before contacting with a patient, was between 20 and 40% which is mainly driven by the false safety feeling of wearing gloves, and we recently experienced outbreaks caused by extremely drug-resistant (XDR) *Klebsiella pneumoniae* and *Acinetobacter baumannii* [20,21]. We aimed to investigate the epidemiological consequences of discontinuing VRE screening in such a setting.

## 2. Material and Methods

### 2.1. Setting and Patients

This retrospective observational study was conducted in tertiary care university adult and oncology hospitals in Ankara, Türkiye. These tertiary care hospitals (two distinct hospital buildings in the same campus) include 1040 and 119 beds, respectively. There are six ICUs with 143 beds in the adult hospital; and there is one ICU with eight beds and a hematopoietic stem cell transplantation unit with 16 beds in the Oncology Hospital.

### 2.2. Infection Prevention and Control Measures for VRE

Between January 2006 and January 2013, patients who were admitted to adult hospital intensive care units, i.e., recipients of allogeneic stem cell transplantation or solid organ transplantation and neutropenic patients who were hospitalized in internal medicine wards, were screened via rectal swabs for VRE once a month regularly. If a patient colonized or infected with VRE was detected in any of the wards, weekly rectal screening was started regardless of suspicion of an outbreak.

After January 2013, the Hospital Infection Control Committee decided to discontinue routine VRE screening due to budget problems as well as other logistic problems. Rectal screening on a weekly basis was performed if there was a cluster of VRE infections in any unit. Point prevalence studies were performed to investigate any change in the epidemiology of VRE-colonized patients via rectal screening twice a year between 2015 and 2017. Patients in whom VRE was detected either via rectal swabs or clinical specimens were isolated in a single-bed room or cohorted with another VRE-positive patient. Strict contact precautions were implemented with gowns, gloves, and other personal protective materials. Isolation was terminated after the presence of three negative subsequent rectal swab samples and the eradication of VRE in the clinical samples. Patient rooms were cleaned and disinfected twice daily, and terminal disinfection was performed at least three times before the admitting of a new patient. A formal policy by which to measure the environmental cleaning quality by using a fluorescent marker was introduced to all wards in 2019. Infection control nurses (ICN) visit the ICUs daily and a surveillance programme has been set for almost 30 years. All positive culture reports from the bacteriology laboratory are followed daily for all wards in order to detect the patients who require isolation for multidrug-resistant bacteria infections.

Hand hygiene compliance rates were observed for all occupational groups in intensive care units following the five moments defined by the World Health Organization (WHO) and were reported as percentages for both study periods in the adult hospital. In the oncology hospital, observations were only made in the Oncology Intensive Care Unit, which started operating in 2015. Therefore, hand hygiene compliance rates were reported for the oncology hospital between 2015 and 2021.

### 2.3. Definitions

VRE-BSI was defined as hospital-acquired if the blood cultures were performed ≥48 h after hospital admission. The incidence density of BSI was calculated per 1.000 patient days. A subsequent episode of VRE-BSI which was detected in the same patient 14 days after the first episode was considered as a new episode. Clustering was defined if VRE was detected in two or more patients in a week regardless of type of positive clinical sample. If these genotypes of the isolates could not be distinguished through arbitrarily primed polymerase chain reaction (AP-PCR) and/or pulsed-field gel electrophoresis (PFGE), it was classified as an outbreak. The data were divided into two periods: a baseline period between January 2006 and January 2013, when routine VRE screening cultures were performed in ICUs and special units; and a second period between January 2013 and January 2022, when routine VRE screening was discontinued. In the second period, VRE screening was performed if there was an outbreak or cluster in any ward or ICU. Weekly screening was stopped after no detection of a new VRE case either via rectal swab cultures or any other cultures in the subsequent two weeks.

### 2.4. Bacterial Isolation and Molecular Analysis

During the study period, blood cultures were performed at bedside and cultured by using the BACT/ALERT 3D automated blood culture system until November 2017 and the BACTEC FX automated blood culture system (Becton Dickinson, Cockeysville, MD, USA) between November 2017 and January 2022. Species identification was performed by using the BD Phoenix system (Becton and Dickinson Diagnostic Systems, Sparks, MD, USA) until 2013. After 2013, species identification of *Enterococcus* spp. was achieved via matrix-assisted laser desorption ionization–time of flight mass spectrometry. Antimicrobial susceptibility testing of *Enterococcus* spp. was performed by using the VITEK 2 system (bioMérieux, Marcy l’Etoile, France) or BD Phoenix system (Becton and Dickinson Diagnostic Systems, Sparks, MD, USA) in the different periods of the study. After December 2017, antimicrobial susceptibility tests were performed by using the BD Phoenix system. Minimum inhibitory concentrations (MICs) of vancomycin and teicoplanin were confirmed via gradient test (BioMérieux). For antimicrobial susceptibility testing, our bacteriology laboratory switched from the Clinical and Laboratory Standards Institute (CLSI) guideline to the European Committee on Antimicrobial Susceptibility Testing (EUCAST) criteria in September 2016 in concordance with European countries. Therefore, in the present study, the results of antimicrobial susceptibility tests were interpreted according to the CLSI recommendations between 2006 and September 2016 [22]. After September 2016, antimicrobial susceptibilities were evaluated according to the EUCAST guideline [23]. *E. faecalis* ATCC 29212 and *E. faecalis* ATCC 51299 were used as quality control reference strains.

Perirectal swabs were transferred to the Infectious Disease Deaprtment Research Laboratory and cultivated in D-coccocel agar (BioMerieux, Craponne, Lyon, France) in which 6 μg/mL vancomycin and 64 μg/mL ceftazidim were added. The plates were incubated in aerobic conditions at 37 °C for 72 h and the bacterial growth was controlled daily. Typical colonies that were Gram-positive were subcultured to tryptic soy agar with 5% sheep blood (BBL, Becton Dickinson) for identification via API 20 STREP (BioMerieux, Craponne, Lyon, France) [24]. The susceptibility testing and determination of minimum inhibitory concentration (MIC) of the *Enterococcus faecium* and *Enterococcus faecalis* isolates were performed via the broth microdilution method prepared in-house according to CLSI (formerly NCCLS) standards [25].

For molecular detection of vanA and vanB genes, the isolates that were grown in pure culture in the medium were taken with a sterile loop and suspended in 1 mL sterile distilled water in 4 McFarland turbidity. They were then boiled at 90 °C for 30 min in a dry heat block (Wealtec Corp., San Francisco, CA, USA). They ware centrifuged at 3000 rpm for 5 min and the resulting supernatants were used as a template DNA sources for amplification. Oligonucleotide primers for the vanA and vanB genes were used in multiplex PCR [26]. PCR was performed on a DNA thermal cycler (Applied Biosystems, Foster City, CA, USA) in a final volume of 50 μL containing 250 ng of DNA as a template, 50 pmol of each oligodeoxynucleotide primer, 200 μM deoxynucleoside triphosphates, 1.5 mM MgCl_2_, and 2 U of Taq DNA polymerase. The PCR products—732 bp for vanA and 635 bp for vanB—were detected via gel electrophoresis in 1.5% agarose with EtBr and visualized under UV light [27].

### 2.5. Molecular Epidemiological Analysis

The strains that were isolated during the possible outbreaks were stored at −80 °C until genotyping. Arbitrarily primed polymerase chain reaction (AP-PCR) and pulsed-field gel electrophoresis (PFGE) were performed as described previously [28]. Briefly, bacterial DNA was extracted using QIAsymphony DSP Virus/Pathogen Kit and the QIAsymphony SP Automated Nucleic Acid Purification System (Qiagen, Helden, Germany) according to the instructions of the manufacturer. The DNA samples were stored at −80 °C until use. The AP-PCR master mix (50 μL) contained 100 ng of template DNA, 100 pmol of M13 primer (5′-GAG GGT GGC GGT TCT-3′), 2.5 U of Taq DNA polymerase (Promega Corporation, Fitchburg, WI, USA), 0.4 mM deoxynucleoside triphosphate mix, 4 mM MgCl_2_, and 10× amplification buffer. Amplification was performed using a Gene Amp PCR System 9700 (Applied Biosystems, Foster City, CA, USA) according to the following conditions: 2 cycles, each consisting of 5 min at 94 °C, 5 min at 38 °C, and 5 min at 72 °C; and 40 cycles, each consisting of 1 min at 94 °C, 1 min at 38 °C, and 2 min at 72 °C. The amplified products were electrophoresed in a 2% agarose gel with ethidium bromide for 1 h at 100 V and 470 min at 50 V and were visualized using the Kodak Gel Logic 200 Imaging System (Eastman Kodak Company, Rochester, NY, USA) under ultraviolet light.

PFGE was performed by using a modified version of the protocol by Denton et al. [16]. Restriction enzyme Xbal (Biolabs, Beverly, CA, USA) was used to cut the chromosomal DNA (8 h at 37 °C). Electrophoresis was performed in 1.2% PFGE agarose on a CHEF DRII apparatus (Bio-Rad, Nazareth, Belgium) with 6 V/cm^2^ for 20 h at 14 °C with an initial switch time of 5 s and a final switch time of 35 s. In addition, the gel was stained with 1 mg/mL ethidium bromide in 0.5× TBE for 30 min. Band profiles obtained via agarose gel electrophoresis were photographed under a UV transducer and stored electronically for analysis.

The band profiles obtained by both genotyping methods were analyzed by using the Gel Compare version 6.6 software program (Applied Maths, Courtrai, Belgium). The Dice Similarity Coefficient was used for band analysis, and the Unweighted Pairwise Grouping Mathematical Averaging (UPGMA) method was used for clustering analysis. The analysis used position tolerance and optimization as 1.0% and 1.0%, respectively. Based on the similarity coefficients of the isolates, strains with over 90% similarity were accepted as indistinguishable clones.

### 2.6. Statistical Analysis

The incidence density rate (IDR) of VRE-BSIs was calculated by using OpenEpi (Open-Source Epidemiologic Statistics for Public Health) version 3.01 software (https://www.OpenEpi.com, 1 October 2022) for each period, and then the density rates of hospital-acquired (HA)-VRE BSIs were compared with each other. The rate of VRE positivity in the periodical rectal screening cultures between 2006 and 2013 was compared with the rate of VRE positivity in the rectal cultures that were taken during point prevalence studies between 2015 and 2017 by using the “two by two table “of OpenEpi. *p*-values < 0.05 were considered statistically significant. Compliance rates of hand hygiene in adult hospital ICUs were compared for each period by using Openepi.

The study was approved by the Hacettepe University Non-Interventional Clinical Research Ethics Committee, Ankara, Turkey (2021/14-53).

## 3. Results

In the adult hospital, 506 out of 10171 (5%) rectal screening cultures were VRE-positive between 2006 and 2013, and 54 out of 569 (9.5%) cultures that were taken during point prevalence studies performed between 2015 and 2017 were VRE-positive (*p* < 0.001). In the oncology hospital, VRE was isolated in 105 out of 1642 (6.4%) of rectal swabs in the first period, and 17 out of 142 (12%) cultures were VRE-positive at point prevalence cultures (*p* = 0.02) (Table 1).

**Figure 1:**
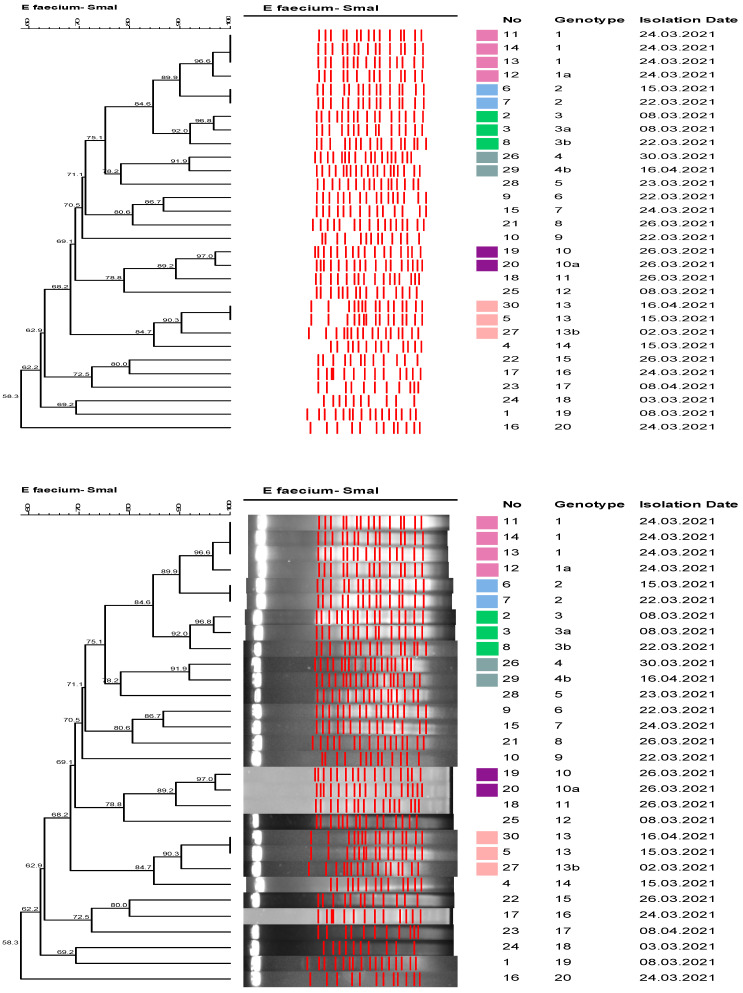
Dendrogram of PFGE Analysis of the VRE Isolates—oncology hospital cluster, 2021 *. * All strains were included in the PFGE analysis.

The IPC team identified two clusters of VRE infections during the second period. The first cluster was detected in the oncology hospital in 2021. After detection of VRE infections in two patients, all wards were screened. VRE was detected in 23 (19.7%) out of 117 rectal swabs and 7 clinical specimens, including 3 urine samples, 2 blood samples from central lines, and pleural and pericardial fluid in each between 2 March 2021 and 16 April 2021. All strains were included in the PFGE analysis, which showed 20 different genotypes. No dominant epidemic clone was detected; 16 of 30 *E. faecium* strains were in any cluster, with a clustering rate of 53% (Figure 1).

**Figure 2:**
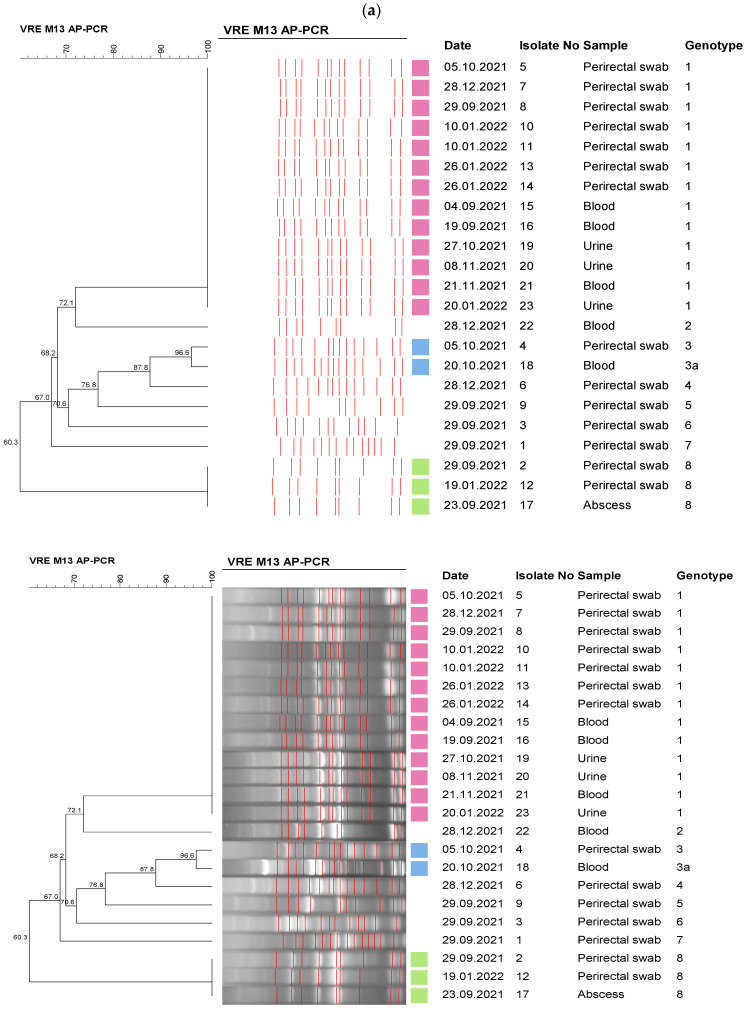

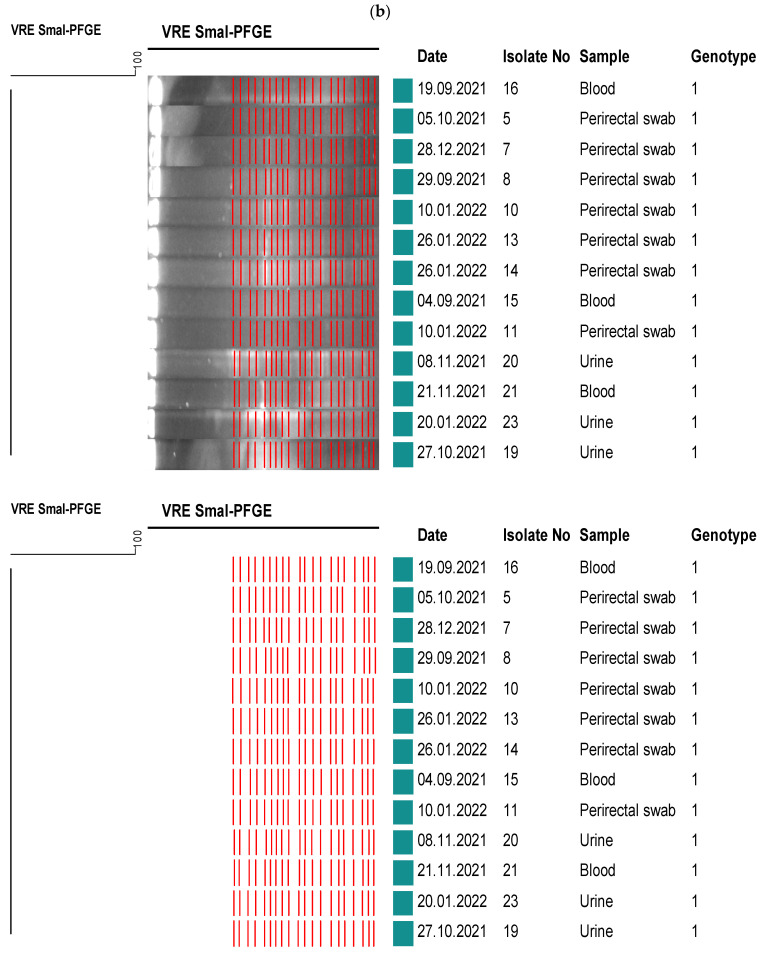
Dendrograms of the VRE isolates—medical ICU outbreak, 2022. (**a**) Dendrogram of AP-PCR analysis. (**b**) Dendogram of PFGE analysis *. * PFGE was performed for the isolates for which a clonal relationship was determined via AP-PCR analysis.

In September 2021, VRE was detected in the clinical samples of two patients hospitalized in the medical ICU in the same week, and then weekly rectal screening was started. VRE was detected in 14 out of the 88 (16%) rectal swabs between September 2021 and January 2022. A total of 9 VRE strains isolated from the clinical specimen of 5 patients and 14 strains that were isolated from rectal swabs were included in the molecular epidemiological investigation. Eight different genotypes were determined among 23 *E. faecium* strains via AP-PCR, and the strains were grouped into three clusters. Then, PFGE typing was performed for 13 isolates in the genotype one cluster, which was thought to be compatible with a possible outbreak. All of the isolates were found to be in the genotype one cluster, and the results were found to be compatible with the AP-PCR (Figure 2).

The VRE clustering in the oncology hospital was not accepted as a single-source outbreak as there were multiple clones; on the other hand, there was a certain clonal outbreak in the medical ICU which was caused by a dominant clone. Increasing the frequency of environmental cleaning and disinfection, cohorting the staff and patients whenever possible, frequent visits by the IPC team, and repeating on-site training to all staff decreased the number of new patients significantly.

The incidence density rates of HA-VRE BSIs before and after the screening policy change are shown in Table 2. There was no statistically significant difference between the two periods in the oncology hospital, the hematopoietic stem cell transplantation (HSCT) unit, the medical ICU, and the surgical ICU. The hospital-wide rates were significantly higher in the second period after discontinuing routine screening in the adult hospital (*p* = 0.015). In order to solve a bias due to the outbreak in the medical ICU, we repeated the analysis after excluding the data from the medical ICU. Then, the incidence density rate decreased from 0.022 to 0.016 in the second period, which was not significantly different from the first period.

The compliance rate for hand hygiene was determined as 45.1% in the first period and 54.6% in the second period in the adult hospital (*p* < 0.01; odds ratio 1.464; confidence interval 95% = 1.434–1.495). Despite an increase in the rates during the second period, they remained low and were found to be below 60% in both periods. On the other hand, in the oncology hospital, the rate was higher at 65.6% between 2015 and 2021.

## 4. Discussion

In our study, the VRE-BSI rate did not increase after discontinuing periodical rectal screening in the adult hospital, which served as general hospital, including patients with COVID-19. When we included the patients hospitalized in the medical ICU in the analysis, there was a trend in the increase in VRE-BSIs; this was the result of an outbreak which we identified via the clinical epidemiology in the early days. Although the patients were hospitalized in single-bed rooms in this ICU, it took two months to control the outbreak. We cannot underestimate the role of weekly screening to monitor the epidemiological changes for VRE-colonized patients; however, despite the fact that weekly screening was started as soon as VRE from was detected in the clinical samples of two patients, unfortunately, the outbreak could only be controlled when colonized patients (as sources of transmission of VRE) passed away or were transferred to other wards.

Herein, a major question raised from our study is the following: if we still had a routine screening protocol, would it be possible to prevent the outbreak? We do not have any record concerning the cumulation of VRE cases during the routine screening period. Routine screening might have the potential to limit the transmission of VRE, but this is difficult to prove without molecular epidemiological investigations on VRE strains in our setting. We experienced only one outbreak after cessation of periodical screening. Even if we accept that weekly screening would prevent an outbreak, we need to take into account the fact that it will require thousands of screening tests and the cost and labor burden that this entails. The annual cost of monthly VRE screening of intensive care units was projected to USD 19,074 from a university hospital in Türkiye [29], and the screening efforts are not reimbursed by the national health insurance company.

The oncology hospital serves patients with a high risk for multidrug-resistant bacteria infections, but discontinuation of periodical rectal screening did not cause an increase in the incidence of VRE-BSIs. Although there was a cumulation of VRE-infected patients in 2021, we did not identify a dominant clone, which suggested that the cumulation was the result of sporadic cases rather than an outbreak.

Our findings encourage the policy of VRE screening via rectal swabs in cases of suspicion of an outbreak. In a study from the USA, the incidence of VRE-BSIs did not change after the discontinuation of active surveillance via rectal screening among patients with hematological malignancies [3]. In another study, a risk-managed approach was implemented to control VRE infections that included infection prevention measures (environmental cleaning) and an antimicrobial stewardship (AMS) program. Routine screening for VRE was discontinued after approximately six months, and the rate of VRE-BSIs remained stable with this VRE risk-managed approach [30].

Cessation of active surveillance and/or contact precautions for VRE did not result in an increase in VRE infection rates in several quasi-experimental studies [14,16,17]. In a single-center study, discontinuation of both contact isolation and active screening was not found to be associated with increased incidence of device-associated infections by VRE [17]. No difference was observed for infection rates in a recent study comparing centers that discontinued contact precautions for VRE/MRSA with centers that continued the standard precautions [31]. In another multicenter study from Canada, changing screening and isolation procedures for VRE did not result in significant adverse clinical outcomes, including all-cause mortality [14].

In contrast to the detection of high VRE colonization rates, not only VRE-BSIs but also overall infection rates were found to be considerably low in a study from Germany that analyzed the results of prospective surveillance and systematic screening. They stopped active screening after the study but continued isolation procedures as a part of their infection control policies, which was similar to our approach in our hospital [32].

In some studies where both screening and isolation practices were discontinued, an increase in the rate of VRE infections was observed [18,19]. In a multicenter study from Canada, 23 hospitals that discontinued contact precautions and routine rectal screening were compared with 77 hospitals that did not change their VRE control policies. An increase in VRE-BSIs was reported from the hospitals which discontinued contact precautions [19]. Centers with low rates of healthcare-associated infections and with high compliance rates of basic infection prevention precautions successfully suspended the isolation precautions in addition to VRE screening [16,17,31]. We believe that centers which did not reach these quality levels, such as our hospital, should still implement contact precautions even if they have discontinued periodical screening.

After our policy change, the average VRE colonization rate was found to be 12% in point prevalence studies between 2015 and 2017. This rate was significantly higher than the rates from the periodical screening period (5%). Although the rate of VRE-BSIs remained similar despite this increase in the colonization rates, the increase in the VRE-colonized patients in point prevalence studies underlines the importance of dynamic monitorization of VRE epidemiology in the hospital.

We followed the incidence rate of VRE-BSIs closely as a part of our surveillance program after discontinuing routine rectal screening and weekly screening of all patients in the wards, where a cumulation of VRE infections continued until there was no detection of any new cases with VRE. Continuous screenings of all patients for VRE on admission and weekly were found to be beneficial during clusters or outbreaks, which was similar to our study [32].

Molecular epidemiology is an essential part of outbreak investigation. We used PFGE and AP-PCR in the investigation of molecular epidemiology. PFGE has been defined as the gold standard for DNA fingerprinting among the several phenotypic and genotypic methods. Although PFGE is the gold standard, PCR-based fingerprinting methods are rapid, cheaper, easier, and reliable for local surveillance [33]. The performances of AP-PCR and PGFE were compatible in our analysis (Figure 2).

Our study had some limitations. First of all, this was a single-center study and results of this study would not be valid for all healthcare settings. Moreover, we are not able to investigate the molecular epidemiology in the period of routine rectal screening and point prevalence studies to identify any possible clonal dissemination. Lastly, colonization with multidrug-resistant bacteria other than VRE through rectal screening was not investigated in this study.

## 5. Conclusions

Active surveillance of VRE via rectal screening is costly and requires a heavy workload. So, VRE screening should be accepted as a guiding tool for an effective VRE surveillance and prevention programme, rather than as the final model for infection preventionists. Therefore, maintaining a costly VRE screening program while barely achieving an average level of basic infection prevention measures seems to be far from providing a significant benefit. In centers that have solved the basic infrastructure, human resources, and cost problems in their IPC programme, the early detection and isolation of patients colonized with VRE can prevent the spread of VRE infections. However, centers that do not have this feature should clearly determine the target of their VRE screening programs. This study demonstrated that the guidance of VRE screening by clinical epidemiology is a realistic, if not ideal, approach in resource-limited centers in terms of IPC programs. In settings where hand hygiene compliance rate is average, such as in our hospital, periodical screening can be discontinued if suspicion of an outbreak can be carefully monitored by the IPC team. Improving compliance to hand hygiene as well as contact isolation precautions are still essential to preventing the transmission of VRE. Molecular epidemiological investigations can give important clues when there is a cluster of patients with VRE infections. Future studies using a cluster randomized controlled crossover design may help to obtain more comprehensible results on the usefulness of VRE screening in non-epidemic conditions as part of the routine IPC programme.

## Figures and Tables

**Table 1:** Comparing VRE-positive rectal screening rates between routine screening and point prevalence results.

	Weekly Screening (VRE-Positive Patients/Number of Screened Patients) (2006–2013)	Point Prevalence Screening (VRE-Positive Patients/Number of Screened Patients) (2015–2017)	*p* Value
Adult Hospital	506/10,171 5%	54/569 9.5%	<0.001
Oncology Hospital	105/1642 6.4%	17/142 12%	0.02

**Table 2:** The incidence density rates of HA-VRE BSIs before and after screening policy change.

	VRE-BSI Rate/1000 Patient Days (95% Confidence Interval) (2006–2012)	VRE-BSI Rate/1000 Patient Days (95% Confidence Interval) (2013–2021)	*p* Value
Oncology Hospital	0.033 (0.014–0.077)	0.042 (0.025–0.072)	0.66
HSCT Unit	0	0.121 (0.047–0.313)	0.06
Hospital-Wide	0.011 (0.006–0.018)	0.022 (0.016–0.031)	0.015
Hospital-Wide Without Medical ICU	0.011 (0.006–0.018)	0.016 (0.01–0.024)	0.22
Medical ICU	0.248 (0.08–0.579)	0.61 (0.382–0.923)	0.06
Surgical ICU	0.3 (0.097–0.7)	0.081 (0.009–0.291)	0.12

HSCT: Hematopoietic Stem Cell Transplantation Unit; ICU: Intensive Care Unit.

## Data Availability

The datasets used and/or analyzed during the current study are available from the corresponding author on reasonable request.
